# CT appearances of Marjolin’s ulcer in the left gluteal region of a young man

**DOI:** 10.2349/biij.2.3.e26

**Published:** 2006-07-01

**Authors:** SI Mohamed, BJJ Abdullah, DA Singh, KS Heng

**Affiliations:** 1 Department of Biomedical Imaging, Faculty of Medicine, University of Malaya, Malaysia; 2 Department of Orthopaedic Surgery, Faculty of Medicine, University of Malaya, Malaysia; 3 Department of General Surgery, Faculty of Medicine, University of Malaya, Malaysia

## Abstract

Chronic wounds and scar tissues are prone to skin cancer. In 1828, Jean-Nicholas Marjolin described the occurrence of tumours in post-traumatic scar tissue. He did not, however, identify the warty ulcers he described as malignant. It was Dupuytren, who about two years later, noted that these lesions were cancerous. The eponym was bestowed by Da Costa in 1903. Marjolin’s ulcer no longer refers only to carcinomas secondary to burns and is classified as a malignancy that arises from previously traumatised, chronically inflamed, or scarred skin. It has been reported in relation to osteomyelitis, venous stasis ulcer, tropical ulcers, chronic decubitus ulcer, frostbite, pilonidal sinus, vaccination site, urinary fistula, hidradenitis suppurativa, skin graft donor site, gunshot wounds, puncture wounds, dog bites, and lupus rash. Early arising Marjolin’s ulcer has rarely been described in literature. In this case report, we present the CT appearances of Marjolin’s ulcer in the left gluteal region of a young man.

## CASE REPORT

A 37-year-old, Chinese man had a 3-month history of intermittent low grade fever and a painful, non-healing ulcer in the left gluteal region. The left gluteal ulcer initially started as a pimple-like swelling 9 years ago. Later, it ruptured and slowly increased in size. The patient had sought treatment from numerous General Practitioners, who only prescribed oral antibiotics and daily dressing. Three months prior to admission, the ulcer begun to enlarge and was increasingly painful. No symptom suggestive of tuberculosis, however, was present. There was no history of discharging sinus. There was also no associated numbness or weakness in the left lower limb. He denied history of weight loss or high risk behaviour, such as, drug abuse or sexual promiscuity. He was not a known diabetic or immunologically compromised. He was also not taking any immune-modulating medication. His past medical history was completely uneventful.

Physical examination revealed a mildly pale, febrile young male with a temperature of 37.5 degree Celsius. Examination of the gluteal region revealed a 4 x 4 cm ulcerated, infected, bad-smelling lesion in the left gluteal fold. The ulcer had a fungating edge with a fixed base and its depth was about 4-5 cm. Mild tenderness was noted on palpation. No neurological deficit was detected in the left lower limb. Palpable left superficial inguinal lymphadenopathy was found consistent with secondary involvement of inguinal lymph nodes. The rest of the systemic examination showed no abnormal findings. Laboratory evaluation revealed mild anaemia with haemoglobin count of 10.4 g/L and a mildly raised white blood cell count of 11,900/ul. The erythrocyte sedimentation rate (ESR) was not elevated. Serology confirmed he was negative for retrovirus as well as Hepatitis B and C viruses. No bacterial cultures were performed from the ulcer here.

**Figure 1:**
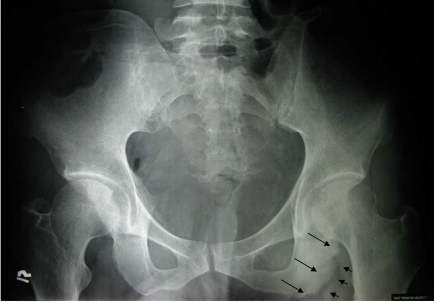
Pelvic radiograph showing area of marked sclerosis, with wide zone of transition and cortical irregularity involving the left ischial tuberosity (thin arrow). Areas of lucencies are seen in the adjacent soft tissue (arrowheads).

**Figure 2:**
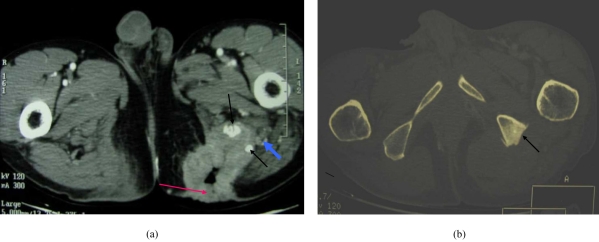
(a) Contrast-enhanced axial CT scan of the pelvis demonstrates an irregular nodular enhancing rim of the ulcer in the left gluteal region, with air pockets and hyperdense foci suggesting bone fragments (short arrow) within it. The sciatic nerve is displaced posteriorly and separate from the tumour (blue arrow); (b) Contrast-enhanced axial CT scan of the pelvis at a higher level (displayed in bone window), showing erosion and sclerosis of the left ischial tuberosity (arrow). There is an associated periosteal new bone formation.

Pelvic radiograph (Figure 1) demonstrated area of marked sclerosis with wide zone of transition and thickening of the left ischial tuberosity. Cortical irregularity was noted, but no calcification was seen. There were also associated lucencies in the adjacent soft tissue. The appearances were suggestive of an aggressive lesion, and the differential diagnoses included chronic osteomyelitis of the left ischial tuberosity with abscess formation and malignancy of the bone. Contrast-enhanced axial CT scan of the thorax, abdomen, and pelvis was performed. This showed a heterogeneously enhancing rim of soft tissue mass in the left gluteus medius and maximus muscles extending inferiorly down to the posterior aspect of the left mid thigh measuring 4.5 x 5.3 x 10 cm (Figure 2). Air pockets and bony fragments were noted within the mass. There were sclerosis and cortical irregularity in the adjacent left ischial tuberosity. Left superficial inguinal lymphadenopathy was noted.

Incisional biopsy of the lesion was performed, and histopathological examination confirmed an invasive, well-differentiated squamous cell carcinoma surrounded by acute inflammation. Wide surgical excision and debridement with distal flap reconstruction and split-thickness skin graft was performed. This confirmed invasion into the ischial tuberosity (which was osteotomised), cartilage, ligaments, and fibromuscular tissue as well as metastasis to the inguinal nodes. The hamstring muscle was not involved and the left sciatic nerve was preserved. The anus and rectum appeared free of tumour involvement. Post-operative recovery was uneventful. Post-operative radiotherapy was given to the primary and draining nodal sites, with a generous margin, because of the ill-defined extension of the tumour. So far, follow-up has not demonstrated any recurrent disease.

## DISCUSSION

While malignancy does not usually arise in ulcers in the developed world, this complication is not rare in developing countries [3]. Marjolin’s ulcers are malignancies that arise at sites of chronic injury. The commonest type of carcinoma arising from Marjolin’s ulcer is SCC, followed by basal cell carcinoma [2]. The male-female ratio is 3:1, and the average age of presentation is 53 to 59 years [3]. Our patient, however, was only 37 years of age. To exclude a primary cancer as the cause of an ulcer, there must be a minimum duration of the lesion of generally 3 years; though others have set this period at 1 month [4]. Our patient, on the other hand, had his ulcer for 9 years. In 40% of the cases Marjolin’s ulcer occurs in the lower extremities, in 30% in the head and neck region, in 20% in the upper extremities, and in 10% in the trunk. Flexion creases are prone to Marjolin’s ulcer [1].

The precise mechanism by which chronic ulcers (wounds) develop malignancy is not known. A variety of causes including chronic irritation and infection (with resulting degeneration and regeneration), decreased vascularity and weakened epithelium, and elevated expression of proto-oncogenes [2] , however, have been suggested for the susceptibility of chronic wounds to malignant transformation. Inflammation, ulceration, and repeated trauma, especially in flexion creases, over many years may provide enough chronic irritation to promote malignant change as in the left gluteal region in our patient. Marjolin’s ulcer is an aggressive epidermoid tumour, and imaging is required only in the cases where deep invasion is suspected.

The essential imaging features of Marjolin’s ulcer are bone destruction, soft tissue mass, and periosteal reaction [2], which were all confirmed in the CT scan of our patient. The soft tissue mass generally appears as irregular and nodular enhancing; with lesion destruction and periosteal reaction in the adjacent bone. Note that plain radiographs may not always demonstrate these changes. MR imaging, due to its superior tissue contrast and multiplanar capabilities, is reported to be better than CT scan at demonstrating the soft tissue mass and the margin and the extent of bone destruction. In addition, it better detects perineural spread along the adjacent nerve [2,6]. In the past, perineural spread was usually diagnosed at surgery or by the pathologist [6]. Our patient did not undergo MRI examination, and his left sciatic nerve was found to be free of tumour during surgery.

Staging of SCCs are based on their size, where T0 lesions are in situ, T1 lesions are less than 2 cm in diameter, T2 lesions are 2-4 cm in diameter, T3 lesions are greater than 4 cm in diameter, and T4 lesions are invasive of muscle or bone [3]. Our patient had a T4 lesion based on this staging. Biopsy is the definitive diagnostic tool and should include tissue specimens from both the centre and margins of suggestive lesions. Simple, punch biopsy usually provides adequate tissue for diagnosis [3].

Preventive care is very important in the management of Marjolin’s ulcer. In all wounds, infection should be treated early, adequate drainage should be provided when necessary, and culture results should be used to choose appropriate antibiotics. In general, recurring ulcers should be excised even if they are not malignant, and skin grafts or flaps should be used for coverage to facilitate complete healing as quickly as possible. Wide local excision, with a margin of at least 1 cm of healthy tissue, should be performed in cases of Marjolin’s ulcer. Regional lymph node dissection is indicated whenever nodes are palpable [3]. Non-surgical treatment, such as applying ionising radiation to the primary or recurrent tumours, is also commonly performed. This is useful for palliation of inoperable primary or recurrent tumours [5].

Long-term follow-up is recommended in all cases of Marjolin’s ulcer because of the possibility of recurrence, metastasis, and other lesions [5]. Most recurrences are regional, but metastases to the brain, liver, lung, kidney, and distant lymph nodes have been reported [3].

## CONCLUSION

Although Marjolin’s ulcers can originate from any long-standing ulcer or region of chronic inflammation, this report has described the unusual development of a metastasising SCC in a chronic, non-healing ulcer in the left gluteal region of a young man. Despite the patient’s young age and apparent good health, the tumour followed an aggressive, metastatic course. Therefore, chronic, non-healing ulcer should be followed carefully to enable the early detection of SCC.
